# Analysis of sequence variability in the *CART *gene in relation to obesity in a Caucasian population

**DOI:** 10.1186/1471-2156-6-19

**Published:** 2005-04-11

**Authors:** Audrey Guérardel, Mouna Barat-Houari, Francis Vasseur, Christian Dina, Vincent Vatin, Karine Clément, Delphine Eberlé, Valérie Vasseur-Delannoy, Christopher G Bell, Pilar Galan, Serge Hercberg, Nicole Helbecque, Natascha Potoczna, Fritz F Horber, Philippe Boutin, Philippe Froguel

**Affiliations:** 1 Institute of Biology-CNRS UMR 8090, Pasteur Institute, Lille, France; 2 University Hospital, Lille, France; 3 Department of Nutrition-EA3502, Paris VI University, INSERM "Avenir" Hôtel-Dieu, Paris, France; 4 Scientific and Technical Institute of Nutrition and Food (ISTNA-CNAM), INSERM U557, INRA U1125, Paris, France; 5 Service d'Epidémiologie et de Santé Publique-INSERM U.508, Pasteur Institute, Lille, France; 6 Dr. Horber Adipositas Stiftung, Hornbachstrasse 50, 8034, Zürich, Switzerland; 7 Imperial College genome Centre and Genomic Medicine, Hammersmith Campus, Imperial College London, UK

## Abstract

**Background:**

Cocaine and amphetamine regulated transcript (CART) is an anorectic neuropeptide located principally in hypothalamus. CART has been shown to be involved in control of feeding behavior, but a direct relationship with obesity has not been established. The aim of this study was to evaluate the effect of polymorphisms within the *CART *gene with regards to a possible association with obesity in a Caucasian population.

**Results:**

Screening of the entire gene as well as a 3.7 kb region of 5' upstream sequence revealed 31 SNPs and 3 rare variants ; 14 of which were subsequently genotyped in 292 French morbidly obese subjects and 368 controls. Haplotype analysis suggested an association with obesity which was found to be mainly due to SNP-3608T>C (rs7379701) (p = 0.009). Genotyping additional cases and controls also of European Caucasian origin supported further this possible association between the *CART *SNP -3608T>C T allele and obesity (global p-value = 0.0005). Functional studies also suggested that the SNP -3608T>C could modulate nuclear protein binding.

**Conclusion:**

*CART *SNP -3608T>C may possibly contribute to the genetic risk for obesity in the Caucasian population. However confirmation of the importance of the role of the *CART *gene in energy homeostasis and obesity will require investigation and replication in further populations.

## Background

Cocaine and amphetamine regulated transcript (CART) is a potent anorectic peptide that is widely expressed in hypothalamic areas and is involved in the control of feeding behavior [1,2]. Immunohistochemistry studies show that CART peptides co-localize with both anorectic and orexigenic hypothalamic peptides [3], particularly with pro-opiomelanocortin (POMC), in the arcuate nucleus (ARC) [4]. Moreover, CART peptides are distributed in peripheral organs notably in the D cells of the endocrine pancreas [5,6] and throughout the peripheral nervous system within the vagal efferent neurons, where it interacts with cholecystokinin [7]. Leptin regulates *CART *mRNA expression, since it is reduced in the arcuate nucleus by the disruption of leptin signaling (in ob/ob mice or fa/fa rats) and increased by leptin peripheral injections [8]. Recent studies have shown that in the context of a high fat diet there is a close relationship between CART and leptin which facilitates the regulation of lipid metabolism in order to control body fat [9]. In rodents, intracerebroventricular injection (ICV) of CART peptide fragments inhibits feeding and antagonizes the feeding response induced by the orexigenic neuropeptide Y (NPY), whereas ICV injection of CART antiserum is found to stimulate feeding [3]. However, instead of the expected hyperphagic phenotype, *CART*-deficient mice are predisposed to obesity only when fed with a calorie-rich diet [10]. Although CART has therefore been shown to be involved in control of feeding behavior, a direct relationship with obesity has not yet been established.

Earlier studies have failed to detect an association between exonic *CART *gene single nucleotide polymorphisms (SNPs) and obesity or obesity-related phenotypes [11-13]. Recently, however, a putative *CART *promoter SNP (-156A>G) has been reported as having a possible association with obesity in a Japanese population [14]. Interestingly, *CART *maps to chromosome 5q13.2, 4.8 Mb from the D5S647 locus, a region previously linked to obesity and serum leptin levels in obese French Caucasian families [15]. *CART *is located 61.8 kb downstream the *MCCC2 *gene (methylcrotonoyl-Coenzyme A carboxylase 2) and is distant to 386 Kb from the *MAP1B *gene (microtubule-associated protein 1B). Only *CART *gene is recognized as candidate for obesity. Recent data have suggested that restricting SNPs analysis to the coding regions only does not adequately describe all the common haplotypes or the true haplotype block structure observed when all of the common variations within the genetic region are used to infer haplotypes [16]. Thus, in this study's investigation of the genetic contribution of *CART *to obesity a region that included the three exons, as well as the two introns and the 5' region from the first ATG to 3.7 kb upstream was screened for SNPs. The initial case-control study was performed in 292 morbidly obese French subjects (BMI ≥ 40 kg/m^2^) and 368 non-obese and normoglycemic controls. In a subsequent extension in the investigation the previously associated SNPs were genotyped in additional sample sets of cases and controls and a haplotype analysis was performed to identify potentially functional SNPs.

## Results

### Initial case-control study

**Figure 1:**
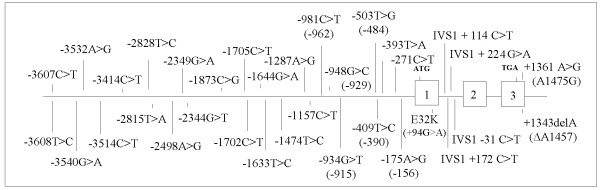
SNPs map of the CART gene. SNPs are reported assigning +1 to the A of the ATG start codon. The numbering reported in the literature is given in parentheses.

**Figure 2:**
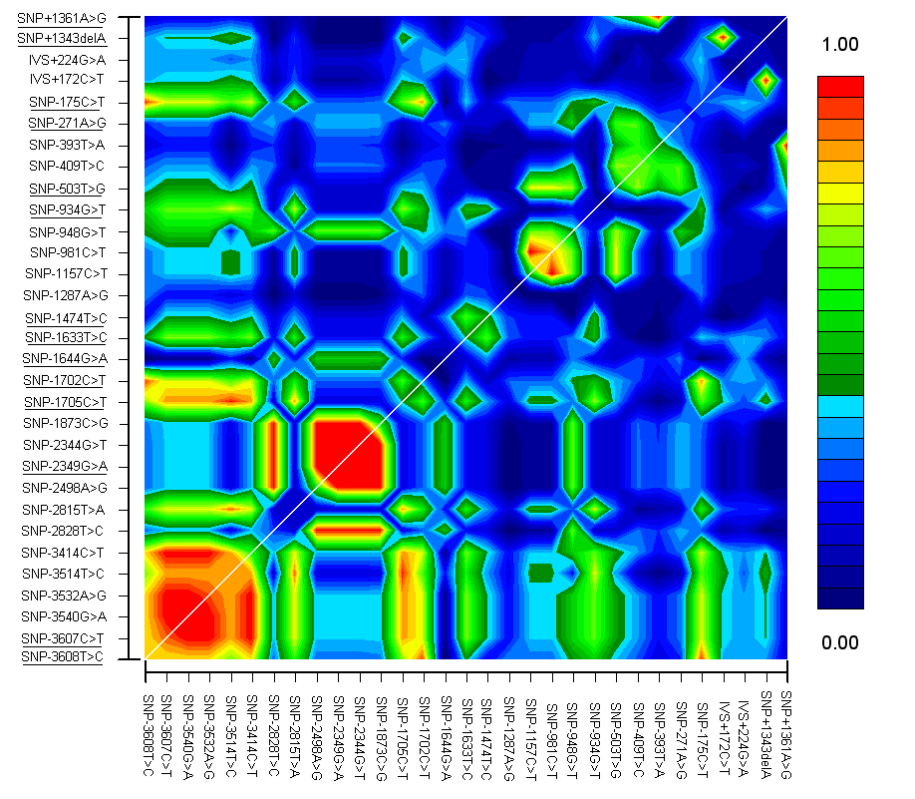
Pairwise linkage disequilibrium between the 31 SNPs of *CART *gene in 45 subjects. The LD was measured by delta (color scale) using the Gold software. SNPs are indicated along the horizontal and vertical axes, according to the first transcribed nucleotide position. Underlined SNPs were selected for the initial case-control study. Regions of high and low linkage disequilibrium are represented by red and blue shading, respectively.

To investigate variation within *CART*, a genetic region of 5.4 kb was sequenced in a total of forty-five subjects (39 obese and six non-obese individuals). A total of thirty-four SNPs were identified (Figure 1) [see Additional file 1]. Three SNPs : a missense Glu32Lys mutation (+94G>A) in exon 1, as well as IVS1+114C>T and IVS1-31C>T both of which resided in intron 1, presented with a frequency lower than 1% (data not shown). None of these rare SNPs were found to co-segregate with obesity or obesity related phenotypes in the probands' families (data not shown). Using the analysis of these thirty-one frequent SNPs (frequency>3%), in this group of forty-five subjects, the linkage disequilibrium (LD) was calculated using the GOLD program (Figure 2). Thus, among the thirty-one SNPs, fourteen SNPs were found to be non-redundant and were subsequently genotyped in 292 morbidly obese subjects and 368 controls. In the 5' region, three SNPs (-3608T>C, -1702C>T and -175A>G) were found to be significantly associated with obesity (*p *= 0.001; *p *= 0.0015; *p *= 0.002 ; see Table 1). A weak association was observed for the 3'UTR SNP +1343delA (*p *= 0.02) and for the 5' SNP-3607C>T (*p *= 0.046). The 14 SNPs were consistent with the Hardy-Weinberg equilibrium (HWE) in the obese group, although in controls, SNP-3608T>C, SNP-3607C>T, SNP-1702C>T, SNP-175A>G deviated from HWE (*p *= 0.0017; *p *= 0.002; *p *= 0.0016 and *p *= 0.0017 respectively). In addition, allele frequency differences between obese and non-obese subjects were confirmed with the Armitage's trend test [17], which doesn't rely on the Hardy-Weinberg equilibrium hypothesis. Nevertheless the genotyping accuracy for these SNPs was subsequently confirmed by duplicate direct sequencing in all those individuals.

**Table 1:** Alleles and genotype distributions for five *CART *SNPs in 368 French non-obese group and in different French morbid obese groups.

**SNPs**	alleles^a^		p-value^b^	OR [95%CI]	genotypes^a^			p-value^c^
**-3608T>C**	**T**	**C**			**TT**	**TC**	**CC**	

French non-obese subjects	311 (42.3)	425 (57.7)			51 (13.9)	209 (56.8)	108 (29.3)	
1st set of French morbid obese subjects	299 (51.4)	283 (48.6)	0.001	1.44 [1.16–1.80]	78 (26.8)	143 (49.1)	70 (24.1)	0.0006
2nd set of French morbid obese subjects	287 (47.5)	317 (52.5)	0.05	1.24 [1.00–1.54]	65 (21.5)	157 (52.0)	80 (26.5)	0.04
Pooled set of French morbid obese subjects*	586 (49.4)	600 (50.6)	0.002	1.33 [1.11–1.61]	143 (24.1)	300 (50.6)	150 (25.3)	0.001

**-3607C>T**	**C**	**T**			**CC**	**CT**	**TT**	

French non-obese subjects	392 (53.3)	344 (46.7)			90 (24.5)	212 (57.6)	66 (17.9)	
1st set of French morbid obese subjects	342 (58.8)	240 (41.2)	0.046	1.33 [1.11–1.61]	102 (35.1)	138 (47.4)	51 (17.5)	0.037
2nd set of French morbid obese subjects	346 (57.1)	260 (42.9)	0.16	1.17 [0.94–1.45]	100 (33.0)	146 (48.2)	57 (18.8)	0.14
Pooled set of French morbid obese subjects	688 (57.9)	500 (42.1)	0.046	1.21 [1.004–1.45]	202 (34.0)	284 (47.8)	108 (18.2)	0.04

**-1702C>T**	**C**	**T**			**CC**	**CT**	**TT**	

French non-obese subjects	438 (63.3)	254 (36.7)			125 (36.1)	188 (54.3)	33 (9.6)	
1st set of French morbid obese subjects	306 (54.4)	256 (45.6)	0.0015	1.44 [1.15–1.81]	82 (29.2)	142 (50.5)	57 (20.3)	0.0009
2nd set of French morbid obese subjects	345 (57.7)	253 (42.3)	0.04	1.27 [1.01–1.58]	94 (31.4)	157 (52.5)	48 (16.1)	0.028
Pooled set of French morbid obese subjects	651 (56.1)	509 (43.9)	0.002	1.35 [1.11–1.64]	176 (30.3)	299 (51.6)	105 (18.1)	0.0015

**-175A>G**	**A**	**G**			**AA**	**AG**	**GG**	

French non-obese subjects	294 (42.4)	400 (57.6)			48 (13.8)	198 (57.1)	101 (29.1)	
1st set of French morbid obese subjects	279 (51.1)	267 (48.9)	0.0022	1.42 [1.13–1.78]	72 (26.4)	135 (49.4)	66 (24.2)	0.0014
2nd set of French morbid obese subjects	289 (46.8)	329 (53.2)	0.10	1.19 [0.96–1.49]	69 (22.3)	151 (48.9)	89 (28.8)	0.095
Pooled set of French morbid obese subjects	568 (48.8)	596 (51.2)	0.007	1.30 [1.07–1.57]	141 (24.2)	286 (49.1)	155 (26.7)	0.006

**+1343delA**	**A**	**delA**			**AA**	**AdelA**	**delAdelA**	

French non-obese subjects	656 (92.7)	52 (7.3)			304 (85.9)	48 (13.6)	2 (0.5)	
1st set of French morbid obese subjects	520 (89.1)	64 (10.9)	0.02	1.53 [1.06–2.28]	232 (79.4)	56 (19.2)	4 (1.4)	0.02
2nd set of French morbid obese subjects	590 (89.6)	68 (10.4)	0.05	1.45 [1.00–2.12]	263 (80.0)	64 (19.4)	2 (0.6)	0.048
Pooled set of French morbid obese subjects	1110 (89.4)	132 (10.6)	0.017	1.50 [1.07–2.10]	495 (79.7)	120 (19.3)	6 (1.0)	0.017

^a^Number of alleles or genotypes (frequencies, %)

^b ^p-value corresponds to association analysis between different French morbidly obese groups (BMI ≥ 40 kg/m^2^) and non-obese subjects (BMI<27 kg/m^2^).

c p-value is calculated by the Armitage's trend test.

**"Risk" **alleles underlined are those encountered more frequently in obese groups.

* Pooled set of 621 French morbidly obese subjects were composed of the first set of 292 French morbidly obese patients (BMI = 47.5 ± 7.8 kg/m^2^, age = 44.7 ± 10.8 years) and of a second set of 329 French morbidly obese patients (BMI = 48.1 ± 7.1 kg/m^2^, age = 48.5 ± 10.5 years).

The genotyping of the five SNPs potentially associated with obesity (5' SNPs : -3608T>C, -3607C>T, -1702C>T, -175A>G and 3'UTR SNP: +1343delA) was then extended to an additional set of 329 morbidly obese subjects. This second French population was not significantly different in mean age or BMI in comparison to the first and therefore enabled pooling of the two case cohorts and the comparison of the SNP allele frequencies between a total of 621 morbidly obese subjects and 368 controls. The -3608T, the -3607C, the -1702T, the -175A and the +1343delA alleles were still more frequent in the morbid obese subjects than in the controls : OR = 1.33, 95%CI = [1.11–1.61], *p *= 0.002 ; OR = 1.21, 95%CI = [1.004–1.45], *p *= 0.046 ; OR = 1.35, 95%CI = [1.11–1.64], *p *= 0.002 ; OR = 1.30, 95%CI = [1.07–1.57], *p *= 0.007 and OR = 1.50, 95%CI = [1.07–2.10], *p *= 0.017 respectively, see Table 1). The results with the most significant p-value of 0.002, which was observed for the SNPs -3608T>C and -1702C>T, was only reached seven times on 1000 permutations, confirming the strength of this result, even though multiple testing was performed.

### Haplotype analysis

The fourteen SNPs were further submitted to haplotype analysis. Rare haplotypes (frequencies < 0.02) were removed. Considering 94.4 % of the existing haplotypes, seven SNPs were identified, which are structured in two combinations of six SNPs (SNPs -3608T>C/ -1705C>T/ -1702C>T/ -1474T>C/ -271C>T/ +1343delA) and (-175A>G/ -1705C>T/ -1702C>T/ -1474T>C/ -271C>T/ +1343delA) represented the haplotype information the best. Haplotype frequencies were compared between the 292 morbidly obese subjects and the 368 controls using the THESIAS (*Testing Haplotype Effects in Association Studies*) software [18]. Both combinations displayed association with obesity (*p *= 0.0003). Then, investigating the effect of individual or group of SNPs on the general haplotypic model, it was observed that the significant effect on obesity was mainly due to i) two combinations of promoter SNPs : (-3608T>C/ -1702C>T) and (-1702C>T/ -175A>G) (*p *= 0.0002 for both combinations) and ii) the presence of the SNP+1343delA (*p *= 0.002). The two combinations (-3608T>C/ -1702C>T/ +1343delA and -1702C>T/ -175A>G/ +1343delA) were analyzed in the 621 morbidly obese subjects and in the 368 controls and were found to show an association with obesity (global *p *= 6.10^-5^). The haplotypes including SNPs -3608T (or -175A), -1702T, +1343A alleles or -3608C (or -175G), -1702C, +1343delA were more frequent in obese subjects than in controls (10.9% vs. 7.2%, OR = 1.91, 95%CI = [1.18–3.12], *p *= 0.009; 44.0% vs. 35.6%, OR = 1.60, 95%CI = [1.26–2.03], *p *= 0.0001 respectively).

These analyses were then implemented in the COCAPHASE program to exclude putative errors in haplotype determination induced by the Hardy Weinberg disequilibrium observed in the controls, which confirmed the results obtained with THESIAS (data not shown). Altogether, these data highlighted the role of SNPs -3608T>C (or -175A>G), and -1702C>T combined to SNP+1343delA in the SNP haplotype structure associated with obesity in French Caucasians.

### Study of SNPs -3608T>C, -1702C>T,-175A>G and +1343delA in a French general population

**Figure 3:**
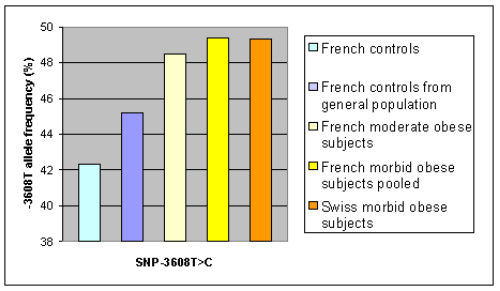
Histogram summarized the -3608T allele frequency in different groups of controls and obese subjects. Compared to French controls from type 2 Diabetes pedigrees and French controls from general population ; -3608T allele frequency was more frequent in the French moderate obese subjects (p = 0.011 and p = 0.14, respectively) and in the Swiss morbidly obese subjects (p = 0.002 and p = 0.078, respectively).

In order to support further our initial findings, we analyzed an independent control group consisting of 732 non obese subjects ascertained from the general French population. All SNPs did not deviate from HWE in this group. When this control group was compared to the 621 French morbid obese subjects, SNP-3608T>C showed a nominal association with morbid obesity (OR = 1.18, 95%CI = [1.01–1.39], *p *= 0.038), although the SNPs -1702C>T, -175A>G and +1343delA were not found to be associated with obesity (data not shown). The prevalence of the SNP -3608C>T T allele was 49.4% in morbidly obese subjects, 45.2% in controls derived from the French general population and 42.3% in the non obese and normoglycemic subjects (Figure 3).

### Study of SNP-3608T>C in morbid Swiss and moderate French obese subjects

To further investigate the impact of the 3608T>C *CART *SNP on obesity in populations of European origin, we extended the study to 619 additional French subjects with moderate obesity (30 kg/m^2^<BMI<40 kg/m^2^) and to 385 morbidly obese Swiss subjects (BMI>40 kg/m^2^). As shown in Figure 3, no significant difference in the T allele frequency was found between the two obese groups or with the initial 621 morbidly obese subjects, thus confirming the high prevalence of this allele in association with obesity. When the three obese group (n = 1,625) and the two control groups (n = 1,100) were analyzed together, an association with obesity and the *CART *3608T>C T allele was confirmed (OR = 1.22, 95%CI = [1.09–1.37], *p *= 0.0005).

### Electrophoretic mobility shift assays (EMSAs) analysis

**Figure 4:**
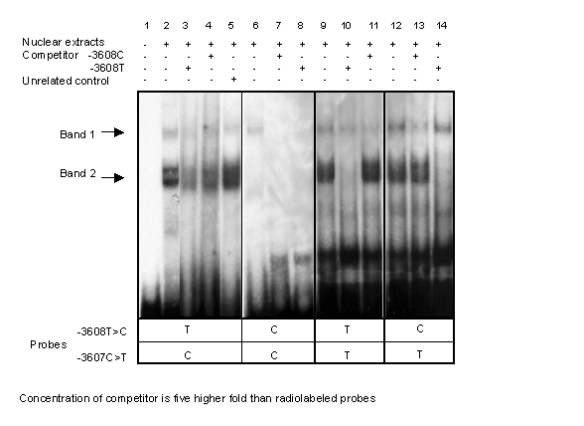
Electrophoretic mobility shift assays for SNP-3608T>C with nuclear extracts prepared from RIN-1027-B2 cells. Four probes used for EMSA contained respectively -3608T-3607C, -3608C-3607C, -3608T-3607T and -3608C-3607T. Specific complex formations (compared lines 2 and 5) are indicated by two arrows. Lane 1 is radiolabeled probes without nuclear extract. In the presence of the -3608T allele and the -3607C allele, two bands (bands 1 and 2) were observed corresponding to the fixation of two different factors (lane 2). The intensity of the band 2 decreased when either of these two alleles was changed (lanes 6, 9 and 12 compared to lane 2). The band 2 decreased by 1.5 fold in a TT or a CT configuration (lanes 9 and 12) and completely disappeared in a CC configuration at both loci (lane 6). The intensity of the band 1 and 2 was decreased when non radioactive competitors (either C or T alleles at the -3608 locus) were added to the reaction (lanes 3 and 4 compared to lane 2). In the TT configuration at the two loci, the addition of the -3608C non labeled probe did not decrease the band 2 signal unlike the -3608T probe (lanes 10 and 11 compared to lane 9). These observations suggest that the band 2 corresponds to the binding of a putative nuclear protein when both the -3608T and the -3607C alleles are present. The band 1 was observed when at least one C allele was present at either locus, decreased by 2 fold in a TT configuration (lanes 2, 6, 12 compared to lane 9).

Therefore genetic studies suggested an association between *CART *SNP-3608C>T and obesity. These data then promoted the investigation of this polymorphism's potential functional effect. Potential effects of plausible promoter SNP -3608T>C on binding affinity for nuclear proteins was evaluated by electrophoretic mobility shift assays (EMSA) in RIN-1027-B2 cells. This cell line was chosen because *CART *expression has been localized in the somatostatin producing islet D pancreatic cells. As SNP -3607C>T is adjacent to SNP -3608T>C, two pairs of DNA oligonucleotide probes were required. In the presence of -3607C, RIN-1027-B2 nuclear proteins (Figure 4, band 2) have a lower affinity for the C allele than the T allele of SNP-3608T>C (Figure 4, lane 6), indicating that the presence of both the -3608T and the -3607C alleles are required for binding. With at least one C allele present for SNP -3608T>C or SNP -3607C>T, a binding (band 1) was observed and this was found to be then decreased two-fold in the -3608T-3607T configuration (Figure 4, lane 9). To identify which SNP loci correspond to which band, homologous and heterologous competition with non-labeled probes were used in EMSA (Figure 4, lanes 3, 4, 7, 8, 10, 11, 13, 14). The addition of either competitor -3608T or -3608C induced a decrease of the two signals for all configurations.

**Figure 5:**
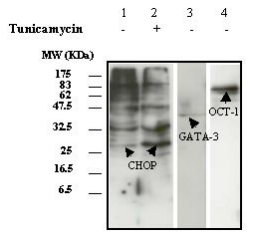
Expression of nuclear proteins in untreated and tunicamycin-treated RIN-1027-B2 cells. GATA-3 and OCT-1 were specifically detected in RIN-untreated cells (lanes 3 and 4). For CHOP, a multiple band pattern was observed (lane 1). As tunicamycin induces endoplasmic stress in cells and consequently enhances CHOP expression, a western blot was performed with tunicamycin treated RIN-1027-B2 cells to determine which band corresponds to CHOP. As pointed by the arrow, the intensity of the band of 27 KDa increased in nuclear extracts from RIN-treated cells, indicating that the amount of CHOP nuclear protein was increased (lane 2 compared to lane 1).

*In silico *analysis  suggested that allele -3608T could be located within at least three putative consensus sequences for transcription factor binding: CHOP/GADD153 (C/EBP homologous protein), GATA-3 and OCT-1. Therefore, the presence of these transcription factors in nuclear extracts from RIN-1027-B2 cells was ascertained by performing western blot analysis with specific antibodies (Figure 5). As expression of CHOP was increased by tunicamycin [19], EMSA experiments were performed on nuclear extracts of treated cells. Results were similar to those presented in Figure 4 (data not shown). Then super-shift assays were performed with anti-GATA-3, anti-OCT-1 antibodies on nuclear extracts from untreated RIN-1027-B2 cells. For anti-CHOP antibody, nuclear extracts were used from tunicamycin-treated and untreated RIN-1027-B2 cells. In the presence of antibodies the patterns of the super-shift assays did not differ from those of the shift assays described in Figure 4 (four replicates, data not shown). These results indicate that none of these three transcription factors seemed to be involved in the constitution of nuclear proteins – DNA complexes including the -3608 locus observed in the EMSA experiments.

## Discussion

This is the first extensive study of the *CART *gene that includes an extensive analysis of the 5' region of the gene (3.7 kb upstream) in association with human obesity. Indeed, Dominguez et al. characterized the region encompassing 3.4 kb of 5' upstream sequence in the mouse CART gene and identified several regulation sites for CART mRNA levels within this region [20]. *CART *is very polymorphic in human : we found thirty-one common SNPs and three rare variants (Figure 1). Of these, the +1343delA (ΔA1457) and +1361A>G (A1475G) located in the 3'UTR have been previously reported in Danish subjects without association with obesity [11]. The +1361A>G (A1475G) SNP was associated with a lower waist-to-hip ratio in British Caucasian non-obese men from the Isle of Ely type 2 diabetes cohort [12], but in our French population showed no association with either obesity or obesity-related phenotypes. In contrast to Fu et al [21], no association between any *CART *SNPs and obesity-related quantitative traits, like lipid levels, was detected in our obese population (data not shown). Six SNPs in the 5'flanking sequence covering a region of 1.1 Kb have been reported in Japanese obese subjects [14]. Instead of the -175G (-156G) allele being associated with obesity in the Japanese obese population, the -175A (-156A) wild type allele was more prevalent in obese than in non-obese French subjects. This discordant result between these two populations might be explained by ethnic differences or a particular environmental influence. Alternatively, it may only show that this SNP is not functional, as suggested by the EMSA data that showed no protein-binding at this site (data not shown). Nevertheless, discordance in allelic frequency between Caucasian and Japanese populations has been previously observed for other diabesity susceptibility genes, such as adiponectin/APM1 [22].

Our study of the genetic variability of the *CART *gene in various obese and control populations of European origin suggests a possible association with obesity which is mainly due to the effect of SNP -3608 T>C. We are aware that the existence of Hardy-Weinberg disequilibrium (in the first cohort of 368 French controls only) may lead to an incorrect estimation of haplotype frequencies and thus of the haplotype effect on obesity. Therefore we used haplotype reconstruction algorithms that rely on the hypothesis of random gamete association minimizing the possible errors induced by absence of HWE. The deviation from Hardy Weinberg equilibrium observed may have several explanations. A random chance may account for deviation from HWE in 1 out of 20 markers (5%) [23]. In these present data, 50% of genotyped markers from the 5' sequences of *CART *deviated from HWE thereby excluding this hypothesis. The full replication of the initial SNP genotypes by direct sequencing makes significant genotyping errors unlikely. This deviation is also unlikely to be due to hidden population structure since an excess of heterozygote subjects was observed, whereas the effect of population structure usually increases homozygote frequency in the whole population [24]. To test whether departure from HWE is specific to *CART*, we reanalyzed previous genotyping data obtained in the 368 controls for eight genes, some of them associated with obesity or associated phenotypes such as UCP3, APM1, PGC1 and GAD2 genes. No deviation from HWE was observed for any of the 27 SNPs from these genes [[22,25,26] and unpublished data]. This would suggest a specific deviation of HWE in the *CART *gene in the non-obese subjects of set 1. Deviation from HWE may also be interpreted as a signal of association [23,27]. Unlike the 732 controls who are representative of the French general population, the initial cohort of 368 non-obese individuals resulted from a selection of non-obese normoglycemic subjects chosen from French type 2 diabetes pedigrees. These subjects live in families with type 2 diabetes where obesity is frequent, and may therefore represent subjects more "resistant" to both type 2 diabetes and obesity in spite of a shared common environment with their diabetic and/or obese family members. The more significant association of SNPs -3608T>C with obesity in this population compared to the more general control population advocates the interest of such a sample for an association study [28]. In addition, the more robust association with morbid obesity highlights that the probability to detect genetic mutations influencing BMI is higher in an obese population with a more extreme phenotype.

EMSA experiments on SNP -3608T>C showed a functional difference between the two alleles at the locus where DNA variation modulates binding affinity in RIN-1027-B2 pancreatic cells. However, we cannot clearly ascertain the functional role of the SNP -3608T>C, since the nucleotide at position -3607 seems to interact with the binding of nuclear factors. Moreover, haplotype analysis comparing the 368 French controls and the 621 French morbidly obese subjects, showed that the -3608T/-3607C combination is associated with obesity (p = 0.0013). Therefore, from our genetic and functional results, we hypothesize that these two SNPs, located one base pair apart, jointly modulate the binding of the same nuclear factor on *CART *gene 5' sequences. Nuclear factors involved in the protein DNA complexes remain to be identified. It also must be considered that functional study in this cell line may or may not reflect all relevant conditions in brain. So, cells from hypothalamic neurons would allow confirmation of this observation and a better understanding of plausible gene regulation.

## Conclusion

This result concludes a possible modest contribution of variation in the CART gene to the genetic susceptibility to obesity. Replication in further populations will be required to further strengthen this association.

## Methods

### Subjects

According to the previously reported linkage results at the 5q locus [15], we selected 45 subjects (39 obese and six non-obese subjects) in families that contributed to the linkage with obesity. The association study was performed using at first a set of 368 non-obese and normoglycemic subjects ([mean ± SD] age, 57.1 ± 13.5 years, BMI = 22.9 ± 2.3 kg/m^2^, sex ratio : women/men 221/147) and a set of 292 morbidly obese French individuals (age, 44.7 ± 10.8 years, range 24 to 74, BMI = 47.5 ± 7.8 kg/m^2^, sex ratio : women/men 232/60). SNPs showing a significant association were genotyped in an extended set of 329 morbidly obese French subjects (age, 48.5 ± 10.5 years, range 24 to 74, BMI = 48.1± 7.1 kg/m^2^, sex ratio : women/men 229/100) from the same population as the first set of morbidly French obese subjects were extracted from. The 385 severely obese subjects from Zurich, Switzerland that were studied were consecutive, unrelated, Caucasian subjects ([mean ± SEM] age, 43.5 ± 0.5 years, range 24 to 69; BMI = 43.4 ± 0.4 kg/m^2^; sex ratio : women/men 302/83) referred to the ? clinic for refractory obesity from January 1999 to December 2000; informed written consent was obtained [29]. The set of 619 moderately obese French subjects were characterized as following: age 49.9 ± 13.4 years, range 24 to 88, BMI = 34.5 ± 2.9 kg/m^2^, sex ratio: women/men 354/265. Two additional sets of control subjects from the French general populations were studied : 546 subjects from the SUVIMAX population [30] ([mean ± SD] age 55 ± 6 years, BMI = 22 ± 1.8 kg/m^2^, sex ratio women/men 246/300) and 186 subjects from the WHO-MONICA Lille population [31] ([mean ± SD] age, 60.6 ± 3.1 years, BMI = 24.7 ± 2.9 kg/m^2^, sex ratio : women/men 98/88). Further analysis was performed by pooling these data.

### Screening and SNP map of the CART gene

We screened for SNPs in 3.7 kb of the plausible promoter region, as well as the exons and the introns of the *CART *gene by direct sequencing. PCR primers and annealing temperatures are available from authors on request. The protocol was carried out using the 96 capillary ABI PRISM^® ^3700 DNA Analyzer (Applied Biosystems, Foster City, CA) with the Big Dye Terminator Cycle Sequencing Ready Reaction Kit, as previously described [32]. SNP positions were assigned according to the A of the translation initiation codon (ATG ; Figure 1). The list of identified SNPs and corresponding rs numbers are presented in the additional file (Table 2).

### Genotyping

Several genotyping methods were used. E32K (+94G>A), IVS1+114C>T, IVS1+224G>A and IVS1-31C>T were genotyped with PCR-RFLP using the Sac1, Blp1, Apa1 and Mae3 restriction enzymes respectively (New England Biolabs). Promoter fragments containing more than two SNPs were genotyped by direct sequencing. All other SNPs were genotyped with the LightCycler™ assay (Roche, Mannheim, Germany) based on hybridization of probes labeled by two different dyes allowing Fluorescence Resonance Energy Transfer (FRET) [33]. A genotyping quality control was performed by introducing duplicates in the PCR plates and by genotyping all individuals twice. Sequences of primers and conditions of LightCycler assays are available on request.

### LD analysis

Pairwise Δ (correlation coefficient between identified *CART *SNPs) was estimated from genotypes for the 45 subjects and the results were visualized by the GOLD program .

### Statistical analysis for association studies

Hardy-Weinberg proportions for cases and controls were tested by the χ^2 ^test. Allelic and genotypic frequencies differences between cases and controls were assessed by χ^2^. A region-wide empirical p-value was calculated through permutation. This involved the individual genotype as a whole and the individual's status being shuffled. This method preserves the correlation between SNPs (LD) while breaking the relation between status and the genotypes. For each replicate or permutation each SNP was tested for association and the most significant p-value was stored. We could then compare this p-value to the best observed p-value.

For the haplotype analysis, identification of the minimal set of SNPs that could account for the genotypic diversity was made by systematic enumeration in each block. Haplotypes frequencies were calculated and, after skipping the haplotypes with a frequency lower than 0.02, each SNP and set of SNPs in turn were removed. Thus we found the SNP combination that preserves the marginal haplotype frequencies. This method is implemented in the STRATEGY software. Effects of haplotype were tested using the THESIAS (*Testing Haplotype Effects in Association Studies*) software. The objective of the program is to perform haplotype-based association analysis in unrelated individuals. This program is based on the maximum likelihood model described in Tregouet *et al. *(2002) and is linked to the SEM algorithm [18]. The effect of haplotypes with a frequency lower than 1% was not included in the analysis. THESIAS allows the simultaneous estimation of haplotype frequencies and of their effects on the phenotype of interest. It is possible to get the log-likelihood of the data under a specific hypothesis concerning haplotype effects by setting some appropriate constraints on regression parameters. The notation β_(*h*) _will refer to the regression parameter characterizing the effect of haplotype *h*. This option is useful for testing for the equality of haplotype effects and to observe the SNP effects on a haplotype. For example, to test the effect of the second SNP among the four existent haplotypes, we could note two equations β_(11) _= β_(12) _and β_(21) _= β_(22)_. If the difference between global likelihood and likelihood for tested SNP is significant, then the SNP tested had an important role in the haplotype combination. Significance of the model was confirmed through permutation with disease. As the Hardy Weinberg disequilibrium observed in controls could induce errors for haplotype analysis, we tested the robustness of this analysis. As a result, the permutation of status among individuals allowed us to confirm the significance of the result. Secondly, the analysis using the UNPHASED/COCAPHASE program [34] on the individuals having unambiguous haplotypes, which does not rely on Hardy Weinberg equilibrium hypotheses was carried out and confirmed a strong association.

### Cell line and treatment

Rat islet somatostatin-producing RIN-1027-B2 cells were grown in Dulbecco's modified Eagles medium (DMEM) supplemented with 10% fetal bovine serum, penicillin (10 U/ml), streptomycin (10 mg/ml) and incubated at 37°C under a 5% CO_2 _atmosphere. To analyse CHOP DNA-binding activity in stressed cells, a set of confluent cells was treated for six hours with 2 μg/ml tunicamycin (Sigma), as previously described [35]. Nuclear extracts were prepared as previously described [36].

### Electrophoretic Mobility Shift Assay (EMSA) experiment and Western blot

For EMSA, protein concentrations were determined by the Bio-Rad protein assay with bovine serum albumin as a standard. Double-stranded DNA probes of 23 bp (forward strand : 5'-gctcactgcaaT/Cctctgccctgc-3') containing the -3608T>C polymorphisms were labeled with T4 polynucleotide kinase using [γ^32^P]ATP, purified with the mini-Quick Spin Columns system (Roche Applied Science, Basel, Switzerland). The labelled probes had a specific activity of ~1 × 10^6 ^cpm/pmol DNA. Binding reactions were performed at least three times to replicate results and in the presence of homologous, heterologous and unrelated control competitors They were carried out in a total volume of 20 μl, containing 35000 cpm of radio labeled probe, 10 μg of nuclear extracts, 2 μg of poly (dI-dC) and a buffer with 20 mM potassium phosphate (pH 7.9), 70 mM KCL, 1 mM DTT, 0.3 mM EDTA and 10% glycerol. Gels were exposed to X-ray films (Kodak, Rochester, New York, United States). Quantitation of the label was performed with the NIH Image software . Nuclear extracts were subjected to SDS-Page, Western blotting and immunolabeling using rabbit anti-CHOP (R-20), rabbit anti-GATA-3 (H-18) or rabbit anti-OCT-1 (C-21) polyclonal antibodies (200 μg/ml) from Santa Cruz Biotechnology, Inc., California, U.S.A. When EMSA was performed with antibodies, the binding mix (listed above) was incubated with antibody for 30 min; where after the radiolabeled probe was incubated for 30 min.

## List of abbreviations

ARC, arcuate nucleus; CART, cocaine and amphetamine regulated transcript; CHOP/GADD153 (C/EBP homologous protein 10); CI, confidence interval; GATA-3, GATA-binding protein 3; OCT-1, octamer-binding transcription factor 1 ; EMSA, electrophoretic mobility shift assay; FRET, fluorescence resonance energy transfer; HWE, Hardy-Weinberg equilibrium; ICV, intracerebroventricular injection; LD, linkage disequilibrium; NPY, neuropeptide Y; OR, odds ratio; POMC, pro-opiomelanocortin; SNPs, single nucleotide polymorphisms.

## Authors' contributions

Audrey Guérardel and Mouna Barat-Houari have screened the *CART *gene for SNPs, have selected the subsequent SNPs for genotyping and genotyped these in the different populations, with the technical help of Vincent Vatin. They have performed most of the statistical analyses (with the technical help of Valérie Vasseur-Delannoy, Francis Vasseur and Nicole Helbecque) under the supervision of Christian Dina. Christopher Bell provided scientific editing of the manuscript. Philippe Froguel has directed the study and Philippe Boutin and Philippe Froguel have directed the redaction of the paper. DNA was provided by Karine Clément, Delphine Eberlé, Pilar Galan, Serge Hercberg, Natascha Potoczna, Fritz F. Horber and Nicole Helbecque.

## Supplementary Material

Additional File 1 List of identified SNPs and rs numbers (UCSC Genome Browser on Human May 2004 Assembly). Click here for file

## References

[B1] Vrang N, Tang-Christensen M, Larsen PJ, Kristensen P (1999). Recombinant CART peptide induces c-Fos expression in central areas involved in control of feeding behaviour. Brain Res.

[B2] Beaudry G, Zekki H, Rouillard C, Levesque D (2004). Clozapine and dopamine D3 receptor antisense reduce cocaine- and amphetamine-regulated transcript expression in the rat nucleus accumbens shell. Synapse.

[B3] Lambert PD, Couceyro PR, McGirr KM, Dall Vechia SE, Smith Y, Kuhar MJ (1998). CART peptides in the central control of feeding and interactions with neuropeptide Y. Synapse.

[B4] Elmquist JK, Elias CF, Saper CB (1999). From lesions to leptin: hypothalamic control of food intake and body weight. Neuron.

[B5] Koylu EO, Couceyro PR, Lambert PD, Ling NC, DeSouza EB, Kuhar MJ (1997). Immunohistochemical localization of novel CART peptides in rat hypothalamus pituitary and adrenal gland. J Neuroendocrinol.

[B6] Jensen PB, Kristensen P, Clausen JT, Judge ME, Hastrup S, Thim L, Wulff BS, Foged C, Jensen J, Holst JJ, Madsen OD (1999). The hypothalamic satiety peptide CART is expressed in anorectic and non- anorectic pancreatic islet tumors and in the normal islet of Langerhans. FEBS Lett.

[B7] Broberger C, Holmberg K, Kuhar MJ, Hokfelt T (1999). Cocaine- and amphetamine-regulated transcript in the rat vagus nerve: A putative mediator of cholecystokinin-induced satiety. Proc Natl Acad Sci U S A.

[B8] Kristensen P, Judge ME, Thim L, Ribel U, Christjansen KN, Wulff BS, Clausen JT, Jensen PB, Madsen OD, Vrang N, Larsen PJ, Hastrup S (1998). Hypothalamic CART is a new anorectic peptide regulated by leptin. Nature.

[B9] Wortley KE, Chang GQ, Davydova Z, Fried SK, Leibowitz SF (2004). "Cocaine- and amphetamine-regulated transcript in the arcuate nucleus stimulates lipid metabolism to control body fat accrual on a high-fat diet.". Regul Pept.

[B10] Asnicar MA, Smith DP, Yang DD, Heiman ML, Fox N, Chen YF, Hsiung HM, Koster A (2001). Absence of cocaine- and amphetamine-regulated transcript results in obesity in mice fed a high caloric diet. Endocrinology.

[B11] Echwald SM, Sorensen TI, Andersen T, Hansen C, Tommerup N, Pedersen O (1999). Sequence variants in the human cocaine and amphetamine-regulated transcript (CART) gene in subjects with early onset obesity. Obes Res.

[B12] Challis BG, Yeo GS, Farooqi IS, Luan J, Aminian S, Halsall DJ, Keogh JM, Wareham NJ, O'Rahilly S (2000). The CART gene and human obesity: mutational analysis and population genetics. Diabetes.

[B13] Walder K, Morris C, Ravussin E (2000). A polymorphism in the gene encoding CART is not associated with obesity in Pima Indians. Int J Obes Relat Metab Disord.

[B14] Yamada K, Yuan X, Otabe S, Koyanagi A, Koyama W, Makita Z (2002). Sequencing of the putative promoter region of the cocaine- and amphetamine-regulated-transcript gene and identification of polymorphic sites associated with obesity. Int J Obes Relat Metab Disord.

[B15] Hager J, Dina C, Francke S, Dubois S, Houari M, Vatin V, Vaillant E, Lorentz N, Basdevant A, Clement K, Guy-Grand B, Froguel P (1998). A genome-wide scan for human obesity genes reveals a major susceptibility locus on chromosome 10. Nat Genet.

[B16] Crawford DC, Carlson CS, Rieder MJ, Carrington DP, Yi Q, Smith JD, Eberle MA, Kruglyak L, Nickerson DA (2004). Haplotype diversity across 100 candidate genes for inflammation lipid metabolism and blood pressure regulation in two populations. Am J Hum Genet.

[B17] Slager SL, Schaid DJ (2001). Case-control studies of genetic markers: power and sample size approximations for Armitage's test for trend. Hum Hered.

[B18] Tregouet DA, Barbaux S, Escolano S, Tahri N, Golmard JL, Tiret L, Cambien F (2002). Specific haplotypes of the P-selectin gene are associated with myocardial infarction. Hum Mol Genet.

[B19] Oyadomari S, Mori M (2004). Roles of CHOP/GADD153 in endoplasmic reticulum stress. Cell Death Differ.

[B20] Dominguez G, Lakatos A, Kuhar MJ (2002). "Characterization of the cocaine- and amphetamine-regulated transcript (CART) peptide gene promoter and its activation by a cyclic AMP- dependent signaling pathway in GH3 cells.". J Neurochem.

[B21] Fu M, Cheng H, Chen L, Wu B, Cai M, Xie D, Fu Z (2002). "[Association of the cocaine and amphetamine-regulated transcript gene with type 2 diabetes mellitus]". Zhonghua Nei Ke Za Zhi.

[B22] Vasseur F, Helbecque N, Dina C, Lobbens S, Delannoy V, Gaget S, Boutin P, Vaxillaire M, Lepretre F, Dupont S, Hara K, Clement K, Bihain B, Kadowaki T, Froguel P (2002). Single-nucleotide polymorphism haplotypes in the both proximal promoter and exon 3 of the APM1 gene modulate adipocyte-secreted adiponectin hormone levels and contribute to the genetic risk for type 2 diabetes in French Caucasians. Hum Mol Genet.

[B23] Lewis CM (2002). Genetic association studies: design analysis and interpretation. Brief Bioinform.

[B24] Hartl DL, Clark AG (1997). Principles of Population Genetics.

[B25] Otabe S, Clement K, Dina C, Pelloux V, Guy-Grand B, Froguel P, Vasseur F (2000). A genetic variation in the 5' flanking region of the UCP3 gene is associated with body mass index in humans in interaction with physical activity. Diabetologia.

[B26] Boutin P, Dina C, Vasseur F, Dubois S, Corset L, Seron K, Bekris L, Cabellon J, Neve B, Vasseur-Delannoy V, Chikri M, Charles MA, Clement K, Lernmark A, Froguel P (2003). GAD2 on Chromosome 10p12 Is a Candidate Gene for Human Obesity. PLoS Biol.

[B27] Nielsen DM, Ehm MG, Weir BS (1998). Detecting marker-disease association by testing for Hardy-Weinberg disequilibrium at a marker locus. Am J Hum Genet.

[B28] Morton NE, Collins A (1998). Tests and estimates of allelic association in complex inheritance. Proc Natl Acad Sci U S A.

[B29] Branson R, Potoczna N, Kral JG, Lentes KU, Hoehe MR, Horber FF (2003). Binge eating as a major phenotype of melanocortin 4 receptor gene mutations. N Engl J Med.

[B30] Hercberg S, Preziosi P, Briancon S, Galan P, Triol I, Malvy D, Roussel AM, Favier A (1998). A primary prevention trial using nutritional doses of antioxidant vitamins and minerals in cardiovascular diseases and cancers in a general population: the SU.VI.MAX study – design methods and participant characteristics. SUpplementation en VItamines et Mineraux AntioXydants. Control Clin Trials.

[B31] Lepretre F, Cheyssac C, Amouyel P, Froguel P, Helbecque N (2004). A promoter polymorphism in CD36 is associated with an atherogenic lipid profile in a French general population. Atherosclerosis.

[B32] Boutin P, Wahl C, Samson C, Vasseur F, Laget F, Froguel P (2000). Big Dye terminator cycle sequencing chemistry: accuracy of the dilution process and application for screening mutations in the TCF1 and GCK genes. Hum Mutat.

[B33] Blomeke B, Sieben S, Spotter D, Landt O, Merk HF (1999). Identification of N-acetyltransferase 2 genotypes by continuous monitoring of fluorogenic hybridization probes. Anal Biochem.

[B34] Dudbridge F (2003). "Pedigree disequilibrium tests for multilocus haplotypes". Genet Epidemiol.

[B35] Wang XZ, Kuroda M, Sok J, Batchvarova N, Kimmel R, Chung P, Zinszner H, Ron D (1998). Identification of novel stress-induced genes downstream of chop. Embo J.

[B36] Schreiber E, Matthias P, Muller MM, Schaffner W (1989). Rapid detection of octamer binding proteins with 'mini-extracts' prepared from a small number of cells. Nucleic Acids Res.

