# Supplementary material for: Analysis of sequence variability in the CART gene in relation to obesity in a Caucasian population
Source: BMC Genet. 2005 Apr 11;6:19. doi: 10.1186/1471-2156-6-19 (PMC1087839; doi:10.1186/1471-2156-6-19)
Supplement: Additional File 1 — List of identified SNPs and rs numbers (UCSC Genome Browser on Human May 2004 Assembly). [file 1471-2156-6-19-S1.pdf]

Table 2 : List of identified SNPs and rs numbers (UCSC Genome Browser on Human May 2004 Assembly)

SNPs	Reference SNP Cluster Report or sequences including SNPs
-3608T>C	rs7379701
-3607C>T	rs7378855
-3540G>A	rs4703649
-3532A>G	rs4704173
-3514C>T	rs4703650
-3414C>T	rs4703651
-2828T>C	rs3761972
-2815T>A	rs10515115
-2498A>G	rs6878082
-2349G>A<br>-2344G>T	ataccatgctagctaaagacagtttataaagtcattctgttcaagatgaa**G>A**aaaa**G>T**cctgtggtat gtaagtgacaaagatgcccctggcttgtttcacaggattt
-1873C>G	rs3763155
-1705C>T	rs3857383
-1702C>T	rs6453132
-1644G>A	rs6859438
-1633T>C	rs3763154
-1474T>C	rs3763153
-1287A>G	rs10515114
-1157C>T	rs3857384
-981C>T	rs4991862
-948G>C	rs3846659
-934G>T	tactctctttctagacaggcttcttggataccaaggggttggtggtgggt**G>T**gggggaggttctggggatg gttaaatttgcctgggcagcctcaaagttca
-503T>G	rs6894603
-409T>C	rs6894758
-393T>A	rs6894772
-271C>T<br>-175A>G	rs17358216<br>atctgtgcgcagagcctcgttcccaggcgcctgga**A>G**cccggcgggcattgacgtcaagcgccggc ggagcgctgcctacagacggt
E32K	ccctgctgctgatgctacctctgttgggtacccgtgcccaggaggacgcc**G>A**agctccagccccgagc cctggacatctactctgccgtggatgatgcctcc
IVS1+114C>T	ccctcctccccccacccccactcctattcccagagtcagggcgcggggag**C>T**tgagcgcaacgcccca ggcacccactgccatccgaagagcgtctcgagctc
IVS1+172C>T	rs11575893
IVS1+224G>A	rs2239670
IVS1-31C>T	ataactagggctggaagtgcgcacctgggctgggctcgcagccaaggcgg**C>T**aacttcaggctccg aagcggtgtgttgcagatcgaagcgctgcaagaagt
1343delA	rs5868607
1361A>G	aagtgcttatgaaggggcgtccattctcctccatacatccccatccctct**A>G**ctttccccagaggaccac accttcctccctggagtttggcttaagcaaca
